# Predictors of treatment with antibiotics and systemic corticosteroids for acute exacerbations of asthma and chronic obstructive pulmonary disease in primary care

**DOI:** 10.1186/s12875-015-0256-3

**Published:** 2015-03-24

**Authors:** Salwan Al-ani, Mark Spigt, Johanna Laue, Hasse Melbye

**Affiliations:** General practice research unit, Department of Community Medicine, UIT Arctic University of Norway, Tromso, Norway; Department of Family Medicine, CAPHRI, Maastricht University, Maastricht, The Netherlands

**Keywords:** COPD, Asthma, Exacerbations, Antibiotics, Systemic corticosteroids

## Abstract

**Background:**

Antibiotic and oral corticosteroid prescribing rate in patients suffering from acute exacerbations of chronic obstructive pulmonary disease (COPD) or asthma in general practice are only sparsely described. Our aim was to identify predictors for such prescribing when results from CRP testing, spirometry, and pulse oximetry are available.

**Methods:**

Patients aged 40 years or more diagnosed with asthma, COPD or both, the previous five years from seven general practice offices in Norway, were invited to a baseline examination and asked to visit their GPs during exacerbations the following 12 months. At all visits, symptoms, chest findings, and results from spirometry, pulse oximetry and CRP testing were registered.

**Results:**

Out of the 376 who took part in baseline examination, 95 patients with an exacerbation were included in the analysis. Based on the diagnosis made by GPs, 46 patients (48.4%) were only registered with asthma, and 49 (51.6%) with COPD (or both diagnosis). 11 patients had taken antibiotics and 16 had taken systemic corticosteroids prior to their visit to their GPs. After excluding those already treated, antibiotics were prescribed in 34.9% and systemic corticosteroids in 42.5% of patients diagnosed with COPD compared to 14.6% and 30.8% respectively in patients only diagnosed with asthma (*P* = 0.02, *P* = 0.2). In the COPD group, antibiotic prescribing was not significantly associated with purulence or other respiratory symptoms, but increased phlegm was a significant predictor of antibiotic prescribing in the whole sample (*P* = 0.04). Prolonged expiration, wheezes and diminished breath sounds also predicted the prescribing of both antibiotics and systemic corticosteroids in the whole sample with *P* values < 0.01. The prescribing rate of antibiotics and systemic corticosteroids also increased with increasing CRP value (*P* = 0.001 and *P* = 0.01, respectively) and with decreasing oxygen saturation (*P* = 0.01 and *P* = 0.003, respectively). FEV_1_/FVC < 0.7 at baseline was as significant predictor in patients with COPD and in the whole sample of patients regarding treatment with antibiotics (*P* = 0.004 and *P* = 0.001, respectively) and treatment with systemic corticosteroids (*P* = 0.004 and *P* = 0.001, respectively).

**Conclusion:**

Chest findings, raised CRP value and decreased oxygen saturation were stronger predictors of prescribing of antibiotics and systemic corticosteroids than were respiratory symptoms. Further evaluation of the importance of these findings to guide treatment of asthma and COPD exacerbations is warranted.

## Background

Chronic obstructive pulmonary disease (COPD) is a huge health problem and it is expected to become an even bigger problem due to the rapidly ageing population worldwide [1]. The course of COPD is usually punctuated by episodes of acute worsening of respiratory symptoms, known as acute exacerbations (AECOPD). Among persons older than 40 years in Norway, spirometry revealed COPD in 18% [2], most of them have mild COPD and not more than 4% are registered with a COPD diagnosis in Norwegian primary care [3]. Patients with asthma also experience exacerbation of their disease, which is characterized by a progressive increase in respiratory symptoms and progressive decrease in lung function sufficient to require a change in treatment [4]. The prevalence of asthma has increased both for children and adults in Norway. It is estimated that 8% of all adults have the disease [5]. In a study on asthma exacerbations, 8.8% of the patients experienced an exacerbation of their asthma during a 3 months period [6]. We have previously reported that in Norwegian COPD patients 46.8% experienced one exacerbation or more in one year [7], similar to the frequency found in an international study [8].

Although both COPD and asthma involves bronchial inflammation and airway limitation, we know that the underlying pathophysiology differs [9,10], but in real life, it is still difficult to differentiate between these diseases [3] and a combination of the two diseases may occur [3,11].

Although the trigger for severe exacerbations in patients with COPD cannot be identified in almost one third of cases, bacterial infection, viral infection and environmental pollutants are triggers in the other two thirds [12] and it is estimated that 50 to 70% of the exacerbations of COPD have an infectious etiology [13]. The standard treatment of AECOPD is usually short-acting bronchodilators, oral corticosteroids and/or antibiotics depending on the presenting symptoms. Antibiotic treatment in patients with AECOPD has been a matter of debate in many years mainly due to difficulties in defining exacerbations and in demonstrating their bacterial etiology. Anthonisen and colleagues divided the exacerbations into three types based on the increase of the three cardinal symptoms: dyspnea, phlegm and purulence with type 1 being the most severe (all three symptoms) [14]. Recent work has shown correlation between sputum purulence and the presence of bacteria [15]. Therefore, the current guidelines recommend antibiotic therapy to Anthonisen type 1 patients and to those with two of the three cardinal symptoms, if increased purulence of phlegm is one of them [13,14,16].

Exacerbations of asthma usually occur in response to an external stimulus (viral upper respiratory tract infection or pollution) and in patients with poor compliance, but can also occur in patients with mild and well-controlled asthma [17]. The assessment of the severity of an asthma exacerbation is usually based on symptoms and physical examination. Lung function and oxygen saturation may also be measured [18]. Correction of hypoxemia, rapid reversal of airway obstruction and reducing the risk of relapse are the main targets for treatment of asthma exacerbations [19]. Short-acting bronchodilators, oral corticosteroids and oxygen, if the patient is hypoxemic, are the main treatment options. Antibiotic therapy is not recommended if the patient does not have pneumonia or other bacterial infections at the same time [18].

More antibiotic prescribing than recommended in COPD guidelines was found in a Dutch study from 2006 [20]. In a cross-sectional study carried out in 6 countries in 2008, Llor et al found that the antibiotic prescribing rate was 78.7% among patients with AECOPD, being higher in patients with type 1 Anthonisen (94.2%), followed by type 2 (84.1%) and type 3 (65.8%). Even patients that did not have any Anthonisen criteria received antibiotics (42.5%) [21]. In all the Anthonisen groups, less antibiotics were prescribed to patients tested with C-reactive protein (CRP).

Due to the risk of microbial resistance development [22] and the side effects of both antibiotics and systemic corticosteroids [23,24], unnecessary use of these treatments should be avoided.

In this study the patients with COPD or asthma exacerbations were, as a routine, tested with the following three biomarkers: CRP test, spirometry and pulse oximetry. We wanted to find out whether the GPs took the results of these tests into account when prescribing antibiotics and systemic corticosteroids, and aimed at determining the predictive value of the biomarkers for such prescribing, also when added to respiratory symptoms and chest signs.

## Methods

This is an observational multicenter prospective cohort study with a baseline registration and a 12 months follow-up period in primary care to investigate the predictors for treatment with antibiotics and systemic corticosteroids in patients with exacerbation of asthma or COPD.

### Setting and participants

Patients from seven general practice offices who were 40 years or older with a diagnosis of asthma or COPD registered in their medical record the previous five years were invited to a baseline study. Out of 380 patients who met between April 2009 and March 2010, 376 were deemed to be in a stable phase of their illness and performed post-bronchodilator spirometry, 210 had been diagnosed with asthma only and 166 with COPD (including 92 with both diagnosis) [3,7]. The GPs recorded whether or not treatment with antibiotics and/or systemic corticosteroids had been prescribed for an exacerbation the previous 12 months, and the patients reported hospitalizations due to exacerbations during the same period. Smoking status and comorbidities were registered. All participants were asked to contact their GP during exacerbations the following 12 months. More details about the baseline registration have previously been reported [7].

The regional committee for Medical and Health Research Ethics in North Norway approved the study. All study participants gave written consent.

COPD exacerbation was defined as an increase in dyspnea, coughing or sputum amount that is acute in onset and beyond normal day-to-day variations, which necessitates a dosage adjustment of medication [12]. Asthma exacerbations were defined as episodes of a progressive increase in shortness of breath, cough, wheezing, or chest tightness or a combination of these symptoms [19]. The patients were asked to consult their GPs within 2-3 days when they experience such an increase in symptoms. For patients who visited the GP with several exacerbations during follow up, the first exacerbation was, as a rule, included in the analysis. A later exacerbation was chosen if the set of data from the first exacerbation was not sufficiently complete.

When the patient consulted during an exacerbation, the GPs registered and graded the patient’s symptoms, chest findings, and the duration of the exacerbation and whether the patient had taken antibiotics or systemic corticosteroids the preceding days. Spirometry, CRP and oxygen saturation were also measured during the consultation.

Spirometry was performed according to the American Thoracic Society/European Respiratory Society guidelines [25], using a Spirare SPS310 Spirometer (Diagnostica AS, Oslo, Norway). The CRP rapid test was carried out using Afinion AS100 Analyzer (Axis-Shield, Scotland), Orion Quickread CRP (Orion Diagnostica Oy, Espoo, Finland), or ABX Micros CRP (HORIBA medical, Montpellier, France), oxygen saturation was measured by a digital handheld pulse oximeter, Onyx II model 0550 (Nonin Medical Inc., Plymouth, MN, USA).

### Statistical analysis

The frequencies of prescribed treatment by patient’s characteristics and clinical findings were analyzed separately in patients only diagnosed with asthma (the asthma group) and in patients diagnosed with COPD or both asthma and COPD (the COPD group). Patients, who had started treatment with antibiotic or oral corticosteroids, respectively, before the consultation, were excluded when predictors of prescribing antibiotic and corticosteroid treatment at the consultation were analyzed. When comparing the prescribing between subgroups, Fisher’s Exact Test was used for 2x2 tables and Chi square statistics when continuous variables had been categorized into more than two categories, usually examining linear-by-linear association. Multivariable logistic regression was done in the whole patient sample with antibiotic prescribing as outcome, adding CRP to relevant symptoms, signs and COPD status at baseline in the explanatory model. Likewise pulse oximetry results were added to symptoms, signs and COPD status in a model with systemic corticosteroid prescribing as outcome. Goodness-of-fit was tested by Hosmer and Lemeshow statistics. Statistical analysis was performed using the SPSS version 19 (IBM, Armonk, NY, USA).

## Results

**Figure 1:**
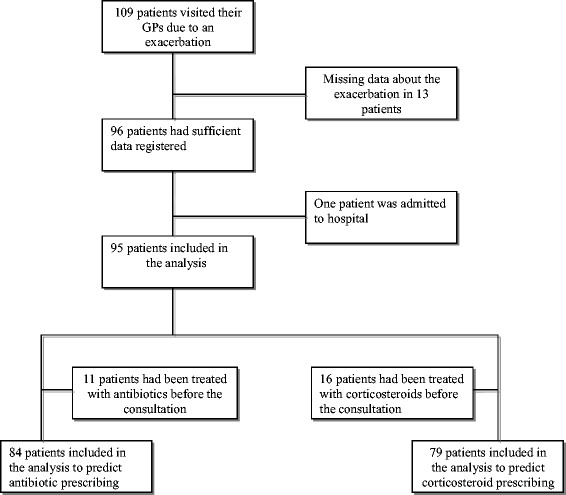
**Flow of patients through our study.**

During the one-year follow-up period, 109 patients visited their GP due to one or more exacerbations. Of these, 14 patients were excluded due to incomplete data, and 95 patients were included in the analysis (Figure 1), 63.2% were female and 47.4% were 65 years or older (mean age 62.1 years). Based on the diagnosis made by the GPs, 46 patients (48.4%) were only registered with asthma, and 49 patients (51.6%) with COPD (or both diagnoses). FEV_1_/FVC < 0.7 post-bronchodilator had been found at baseline in 39 of the patients (41.1%, mean age 65.2 years); whereas 56 patients (58.9%) had FEV_1_/FVC ≥ 0.7 (mean age 59.9 years). Other baseline characteristics are shown in Table 1.

**Table 1:** **Characteristics of the 95 patients taking part in the study**

	***Asthma n*** **(%)**	***COPD/Both n*** **(%)**	***All n*** **(%)**
All	46 (48.4)	49 (51.6)	95 (100)
Age 65 years or more	17 (37.0)	28 (57.1)	45 (47.4)
Gender			
Male	17 (37.0)	18 (36.7)	35 (36.8)
Female	29 (63.0)	31 (63.3)	60 (63.2)
Smoking status			
Never smoker	18 (39.1)	8 (16.3)	26 (27.4)
Current smoker	12 (26.1)	18 (36.7)	30 (31.6)
Ex-smoker	16 (34.8)	23 (46.9)	39 (41.1)
Spirometry at baseline			
FEV_1_/FVC < 0.7	7 (15.2)	32 (65.3)	39 (41.1)
Respiratory symptoms			
Dyspnea, bothersome or very bothersome	42 (91.3)	47 (95.9)	89 (93.7)
very bothersome	12 (26.1)	19 (38.8)	31 (32.6)
Phlegm, bothersome or very bothersome	35 (76.1)	36 (73.5)	71 (74.7)
very bothersome	9 (19.6)	9 (18.4)	18 (18.9)
Purulence	23 (50.0)	21 (42.9)	44 (46.3)
Coughing, bothersome or very bothersome	42 (91.3)	43 (87.8)	85 (89.5)
very bothersome	16 (34.8)	15 (30.6)	31 (32.6)
Chest findings			
Prolonged expiration	16 (34.8)	26 (53.1)	42 (44.2)
Wheezes/rhonchi	16 (34.8)	31 (63.3)	47 (49.5)
Diminished breath sounds	4 (8.7)	15 (30.6)	19 (20.0)
Crackles	10 (21.7)	18 (36.7)	28 (29.5)
Any abnormal chest finding	24 (52.2)	39 (79.6)	63 (66.3)
Admitted to hospital due to exacerbation the year before baseline	3 (6.6)	3 (6.1)	6 (6.4)
Exacerbation treated with antibiotics the year before baseline	13 (28.3)	24 (49.0)	37 (38.9)
Exacerbation treated with systemic corticosteroids the year before baseline	7 (15.2)	24 (49.0)	31 (32.6)

**Figure 2:**
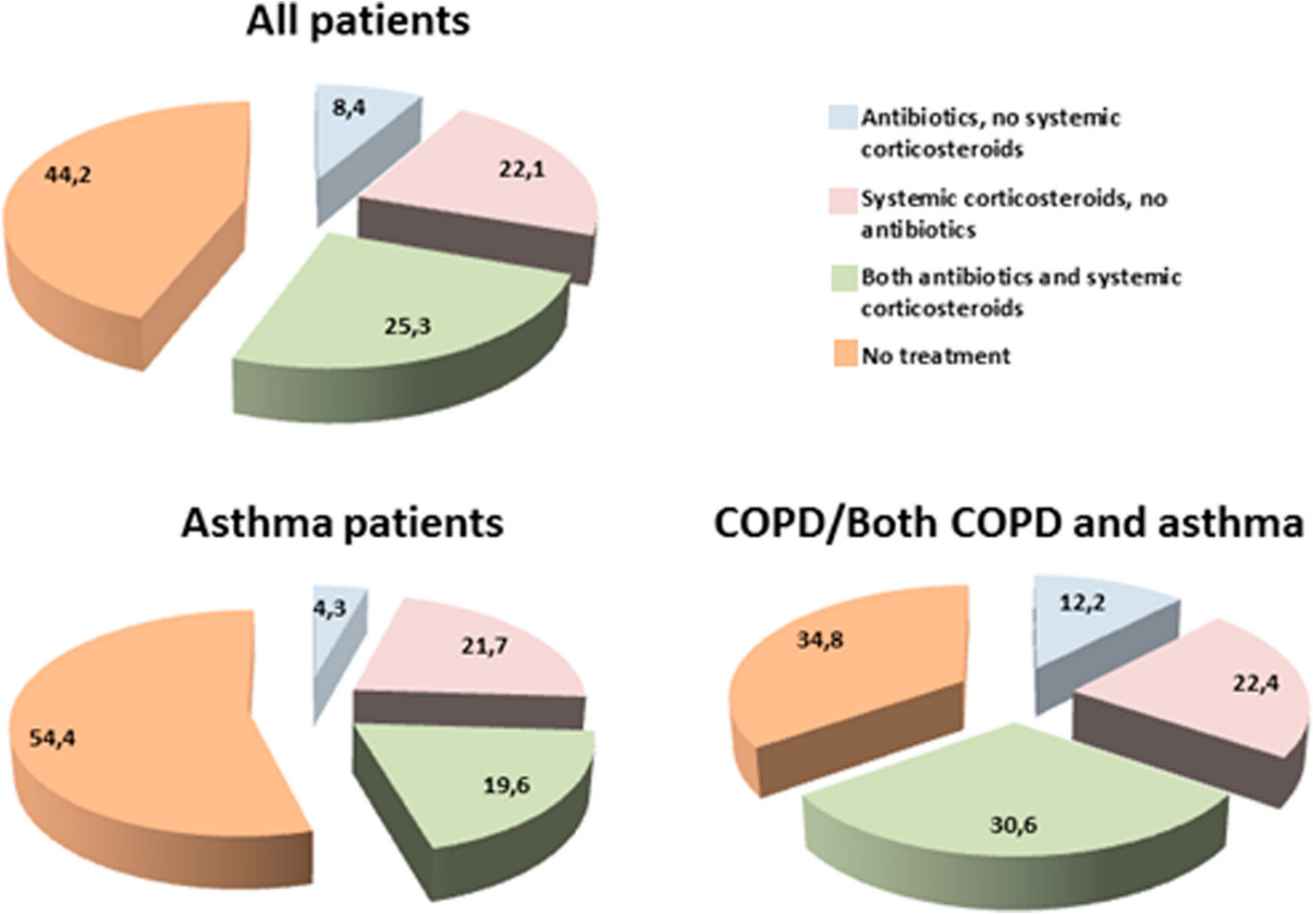
**Percentages of 95 patients treated with antibiotics, systemic corticosteroids or both, which includes the treatment taken before the consultaion.**

During exacerbation, bothersome or very bothersome dyspnea was the most frequent symptom recorded by the GPs in the whole sample of patients whether diagnosed with asthma or not, followed by coughing and phlegm (Table 1). Purulence was recorded in 42.9% in the COPD group and in 50% in the asthma group. Of the chest findings, wheezes and prolonged expiration were most frequently registered. When adding medication taken prior to the consultation, 8.4% of the included patients were treated with antibiotics alone, 22.1% were treated with systemic corticosteroids alone, and 25.3% were treated with both medications (Figure 2). Patients in the COPD group were treated more often with antibiotics and systemic corticosteroids than those in the asthma group (Figure 2).

### Predictors of antibiotic prescribing

Antibiotic prescribing in the whole sample of patients increased with increasing symptom load from 10% in patients with Anthonisen type III, to 28.1% in type II and 31.3% in patients with type I (*P* = 0.1, Table 2). This association was still not statistically significant when the analysis was done separately in the asthma and COPD groups. Among COPD patients with purulence, 33.3% were prescribed antibiotics (*P* = 0.5), whereas the corresponding figure in COPD patients with bothersome or very bothersome phlegm was 40.6% (*P* = 0.2), and phlegm was a significant predictor of antibiotic prescribing in the whole sample (*P* = 0.04). All the chest findings except crackles were significant predictors of antibiotic prescribing in the whole sample of patients (Table 2). The prescribing rate increased with increasing CRP value in the COPD group and in the whole sample of patients as shown in (Table 2). The same trend was found regarding oxygen saturation, with increased prescribing rate in patients with oxygen saturation less than 93%. The strong association between antibiotic prescribing and both baseline FEV1/FVC < 0.7 and raised CRP values were confirmed by the multivariable analysis (Table 3). The *P*-value of the Hosmer and Lemeshow goodness-of-fit test was 0.6.

**Table 2:** **Antibiotic prescribing by patient characteristics for exacerbations of asthma (41 patients) and COPD (43 patients)**

	***Asthma n*** **(%)**	***P-*** **value**	***COPD/Both n*** **(%)**	***P-*** **value**	***All n*** **(%)**	***P-*** **value**
All	6/41 (14.6)		15/43 (34.9)		21/84 (25.0)	
*Baseline characteristics*						
Age						
≥65 years	1/13 (7.7)	0.4	9/25 (36.0)	0.6	10/38 (26.3)	0.5
<65 years	5/28 (17.9)		6/18 (33.3)		11/46 (23.9)	
Gender						
Male	3/15 (20.0)	0.4	5/16 (31.3)	0.5	8/31 (25.8)	0.5
Female	3/26 (11.5)		10/27 (37.0)		13/53 (24.5)	
Smoking status						
Never smoker	2/15 (13.3)	0.6*	2/8 (25.0)	0.7*	4/23 (17.4)	0.8*
Current smoker	3/11 (27.3)		8/16 (50.0)		11/27 (40.7)	
Ex-smoker	1/15 (6.7)		5/19 (26.3)		6/34 (17.6)	
Spirometry						
FEV_1_/FVC <0.7	3/7 (42.9)	0.05	13/27 (48.1)	0.02	16/34 (47.1)	< 0.001
*Characteristics at exacerbation*						
Respiratory symptoms						
Dyspnea, bothersome or very bothersome	6/38 (15.8)	0.6	15/41 (36.6)	0.4	21/79 (26.6)	0.2
very bothersome	2/9 (22.2)	0.4	6/15 (40.0)	0.4	8/24 (33.3)	0.2
Phlegm, bothersome or very bothersome	6/30 (20.0)	0.1	13/32(40.6)	0.2	19/62 (30.6)	0.04
very bothersome	2/7 (28.6)	0.3	4/6 (66.7)	0.09	6/13(46.2)	0.06
Purulence	4/19 (21.1)	0.3	6/18 (33.3)	0.5	10/37 (27.0)	0.4
Coughing, bothersome or very bothersome	6/38 (15.8)	0.6	14/38 (36.8)	0.4	20/76 (26.3)	0.3
very bothersome	2/14 (14.3)	0.7	6/12 (50.0)	0.2	8/26 (30.8)	0.3
Anthonisen criteria combined						
Type III	0/10 (0)	0.08*	2/10 (20.0)	0.4*	2/20 (10.0)	0.1*
Type II	2/15 (13.3)		7/17 (41.2)		9/32 (28.1)	
Type I	4/16 (25.0)		6/16 (37.5)		10/32 (31.3)	
Chest findings Prolonged expiration	4/14 (28.6)	0.09	11/23 (47.8)	0.06	15/37 (40.5)	0.004
Wheezes/rhonchi	4/15 (26.7)	0.1	12/27 (44.4)	0.08	16/42 (38.1)	0.005
Diminished breath sounds	2/4 (50.0)	0.09	7/13 (53.8)	0.08	9/17 (52.9)	0.005
Crackles	2/9 (22.2)	0.4	7/16 (43.8)	0.3	9/25 (36.0)	0.1
Any abnormal chest finding	4/22 (18.2)	0.4	14/34 (41.2)	0.09	18/56 (32.1)	0.03
Lung function^a^						
FEV_1_% predicted < 50	0/1 (0)	0.9*	3/10 (30.0)	0.7*	3/11 (27.3)	0.2*
FEV_1_% predicted 50-80	2/10 (20.0)		10/25 (40.0)		12/35 (34.3)	
FEV_1_% predicted ≥ 80	4/25 (16.0)		1/6 (16.7)		5/31 (16.1)	
C-reactive protein^b^						
<8 mg/L	4/24 (16.7)	0.9*	3/24 (12.5)	<0.001*	7/48 (14.6)	0.001*
8-39 mg/L	2/11 (18.2)		6/10 (60.0)		8/21 (38.1)	
≥40 mg/L	0/1 (0)		5/6 (83.3)		5/7 (71.4)	
Oxygen saturation (SpO_2_)^c^						
>95%	5/27 (18.5)	0.3*	6/23 (26.1)	0.06*	11/50 (22.0)	0.01*
93-95%	0/3 (0)		1/8 (12.5)		1/11 (9.1)	
<93%	1/1 (100)		6/9 (66.7)		7/10 (70.0)	

*P-value calculated using linear-by-linear Chi-Square tests otherwise *P*-value calculated using Fisher’s Exact Test.

^a^7 missing, 5 in asthma group and 2 in the COPD/Both group.

^b^8 missing, 5 in asthma group and 3 in the COPD/Both group.

^c^13 missing, 10 in asthma group and 3 in the COPD/Both group.

**Table 3:** **Predictors for treatment with antibiotics during exacerbations in 84 patients with asthma or COPD using multivariable logistic regression**

	**OR (95%CI)**	***P*** **-value**
FEV_1_/FVC < 0.7 - yes vs no	4.6 (1.3-17.1)	0.02
Bothersome or very bothersome phlegm	3.1 (0.5-18.3)	0.2
Prolonged expiration	2.2 (0.6-8.4)	0.2
C-reactive protein ≥ 8 mg/L - pos vs neg	4.3 (1.3-14.8)	0.02

### Predictors of prescribing systemic corticosteroids

Being 65 years old or more was a significant predictor of prescribing systemic corticosteroids only in patients with asthma, *P* = 0.03 (Table 4). Of the symptoms recorded by GPs, very bothersome dyspnea and coughing predicted the treatment with systemic corticosteroids in patients in the COPD group, but not in the asthma group. Crackles were the only chest finding predicting treatment with systemic corticosteroids in the COPD group (*P* = 0.003), while prolonged expiration and diminished breath sounds were significant in predicting this type of treatment in patients with asthma (Table 4). All the patients with C-reactive protein (CRP) ≥ 40 were treated with systemic corticosteroids regardless diagnosis, and the prescribing rate increased with decreasing oxygen saturation in the COPD group (*P* = 0.004).

**Table 4:** **Systemic corticosteroid prescribing by patient characteristics for exacerbations of asthma (39 patients) and COPD (40 patients)**

	***Asthma n*** **(%)**	***P-*** **value**	***COPD/Both n*** **(%)**	***P-*** **value**	***All n*** **(%)**	***P-*** **value**
All	12/39 (30.8)		17/40 (42.5)		29/79 (36.7)	
*Baseline characteristics*						
Age						
≥65 years	1/13 (7.7)	0.03	10/23 (43.5)	0.5	11/36 (30.6)	0.2
<65 years	11/26 (42.3)		7/17 (41.2)		18/43 (41.9)	
Gender						
Male	3/13 (23.1)	0.4	8/13 (61.5)	0.09	11/26 (42.3)	0.3
Female	9/26 (34.6)		9/27 (33.3)		18/53 (34.0)	
Smoking status						
Never smoker	3/16 (18.8)	0.5*	0/7 (0)	0.2*	3/23 (13.0)	0.1*
Current smoker	5/9 (55.6)		10/17 (58.8)		15/26 (57.7)	
Ex-smoker	4/14 (28.6)		7/16 (43.8)		11/30 (36.7)	
Spirometry						
FEV_1_/FVC < 0.7	3/5 (60.0)	0.1	15/25 (60.0)	0.004	18/30 (60.0)	0.001
*Characteristics at exacerbation*						
Respiratory symptoms						
Dyspnea, bothersome or very bothersome	11/35 (31.4)	0.6	17/38 (44.7)	0.3	28/73 (38.4)	0.3
very bothersome	4/9 (44.4)	0.3	9/12 (75.0)	0.01	13/21 (61.9)	0.006
Phlegm, bothersome or very bothersome	10/28 (35.7)	0.2	14/31 (45.2)	0.4	24/59 (40.7)	0.2
very bothersome	2/7 (28.6)	0.6	5/7 (71.4)	0.1	7/14 (50.0)	0.2
Purulence	7/17 (41.2)	0.2	7/18 (38.9)	0.2	14/35 (40.0)	0.4
Coughing, bothersome or very bothersome	12/35 (34.3)	0.2	17/36 (47.2)	0.09	29/71 (40.8)	0.02
Very bothersome	5/14 (35.7)	0.4	9/13 (69.2)	0.02	14/27 (51.9)	0.04
Anthonisen criteria combined						
Type III	2/11 (18.2)	0.08*	3/8 (37.5)	0.8*	5/19 (26.3)	0.1*
Type II	3/14 (21.4)		7/16 (43.8)		10/30 (33.3)	
Type I	7/14 (50.0)		7/16 (43.8)		14/30 (46.7)	
Chest findings						
Prolonged expiration	7/13 (53.8)	0.03	12/23 (52.2)	0.1	19/36 (52.8)	0.006
Wheezes/rhonchi	7/14 (50.0)	0.06	13/26 (50.0)	0.2	20/40 (50.0)	0.01
Diminished breath sounds	4/4 (100)	0.006	7/12 (58.3)	0.2	11/16 (68.8)	0.004
Crackles	4/8 (50.0)	0.2	12/17 (70.6)	0.003	16/25 (64.0)	0.001
Any abnormal chest finding	9/21 (42.9)	0.08	15/33 (45.5)	0.3	24/54 (44.4)	0.03
Lung function^a^						
FEV_1_% predicted < 50	0/1 (0)	0.8*	5/10 (50.0)	0.08*	5/11 (45.5)	0.1*
FEV_1_% predicted 50-80	4/9 (44.4)		10/22 (45.5)		14/31 (45.2)	
FEV_1_% predicted ≥ 80	7/23 (30.4)		0/6 (0)		7/29 (24.1)	
C-reactive protein^b^						
<8 mg/L	7/21 (33.3)	0.4*	7/21 (33.3)	0.02*	14/42 (33.3)	0.01*
8-39 mg/L	4/11 (36.4)		3/10 (30.0)		7/21 (33.3)	
≥40 mg/L	1/1 (100)		6/6 (100)		7/7 (100)	
Oxygen saturation (SpO_2_)^c^						
>95%	8/24 (33.3)	0.3*	6/23 (26.1)	0.004*	14/47 (29.8)	0.003*
93-95%	1/3 (33.3)		2/6 (33.3)		3/9 (33.3)	
<93%	1/1 (100)		7/8 (87.5)		8/9 (88.9)	

*P-value calculated using linear-by-linear Chi-Square tests otherwise *P*-value calculated using Fisher’s Exact Test.

^a^8 missing, 6 in asthma group and 2 in the COPD/Both group.

^b^9 missing, 6 in asthma group and 3 in the COPD/Both group.

^c^14 missing, 11 in asthma group and 3 in the COPD/Both group.

By entering FEV1/FVC < 0.7 at baseline, very bothersome dyspnea, crackles and pulse oximetry ≤ 92% into multivariate logistic regression model (Table 5), FEV1/FVC < 0.7 and crackles were found to be significant predictors of prescribing. The *P*-value of the Hosmer and Lemeshow goodness-of-fit test was 0.47.

**Table 5:** **Predictors for treatment with systemic corticosteroids during exacerbations in 79 patients with asthma or COPD using multivariable logistic regression**

	**OR (95%CI)**	***P*** **-value**
FEV_1_/FVC < 0.7 - yes vs no	3.7 (1.1-11.7)	0.03
Very bothersome dyspnea - yes vs no	2.9 (0.8-9.9)	0.09
Crackles - yes vs no	4.3 (1.3-14.0)	0.01
Oxygen saturation (SpO_2_) < 93% - yes vs no	5.7 (0.6-56.4)	0.1

## Discussion

### Main findings

Patients diagnosed with COPD or both COPD and asthma by theirs GPs at baseline, were treated more often with antibiotics and systemic corticosteroids during exacerbations than the patients only diagnosed with asthma. Most remarkably we found that the presence of purulence did not significantly predict antibiotic prescribing, not even among the COPD patients, whereas chest finding and biomarkers were significant predictors of the prescribing of both antibiotics and corticosteroids.

### Comparisons with other studies

In our study increased phlegm was the only Anthonisen criterion significantly associated with antibiotic prescribing. In an international study on antibiotic treatment of COPD exacerbation by Llor et al., purulence was the strongest predictor of antibiotics prescribing in countries without access to CRP testing [21]. Where the CRP test was available purulence was still a strong predictor of antibiotic prescribing, but in contrast to our study, CRP testing was not carried out as a routine in all patients. In a British study of patients with lower respiratory tract infection, including patients with AECOPD, antibiotic prescribing was associated with both purulence and abnormal chest findings [26]. Strong emphasis laid on chest findings when prescribing antibiotics has also been reported in other previous studies [27-29].

Llor et al. found that the availability (and use) of CRP testing reduced the weight laid on the presence of purulence [21], similar to that found in a study on patients with acute cough [28]. The use of the CRP in AECOPD was strongly supported in a recent study. A CRP value > 40 mg/L was the strongest predictor of treatment failure in the placebo arm of a clinical trial with AECOPD patients [13]. In Norway, the antibiotic prescribing rate for lower respiratory tract infection is lower than in most European countries [30]. One explanation may be easy access to CRP testing, which has shown to influence the prescribing rate by reducing the unnecessary prescription of antibiotics in patients presenting with acute cough in primary care [31].

Systemic corticosteroids were more frequently prescribed than antibiotics. Although prescribing systemic corticosteroids in the management of exacerbations of COPD or asthma seems to be less controversial than the prescription of antibiotics [12,19], antibiotics have been shown to be the more frequently prescribed of these two medications in a study from Sweden [32]. A greater proportion of our patients had also been treated with antibiotics than with systemic corticosteroids the year prior to the baseline examination. The systematic information from pulse oximetry and CRP test might have contributed to a twist in the treatment in favor of systemic corticosteroids.

Treating asthma exacerbations with systemic corticosteroids is recommended in the current guidelines when short-acting beta 2-agonists do not give sufficient relief [18]. Among the patients only diagnosed with asthma by their GPs, prolonged expiration and diminished breath sounds were the strongest predictors, although not on the list of signs indicating severe asthma attacks in the guidelines from Global Initiative for Asthma (GINA) [18,19].

### Strengths and limitations

As far as we know, this is the first observational study in primary care to investigate the predictors for treatment with antibiotics and systemic corticosteroids including patients with asthma, COPD or both in one patient sample. However, limitations in this study necessitate some caution when interpreting the results. The small number of patients included in the analysis made the results less reliable in particular after dividing the patients into asthma and COPD groups. CRP values ≥ 40 mg/L and SpO2 values ≤ 92 were strongly associated with high prescribing rates of both antibiotics and systemic corticosteroids, and the predictive value of other findings may have been influenced by the co-presence of such findings. For instance, the finding of crackles was significantly associated with low oxygen saturation (*P* = 0.001), and this might have contributed to the high prescribing rate of systemic corticosteroids in the patients with crackles. However, crackles was still a significant predictor of systemic corticosteroid prescribing in the multivariable analysis.

This study does not show what is really going on in daily practice, but what may happen with decisions on prescribing when both CRP testing and pulse oximetry is carried out as a routine. Even when these tests are available they would not have been applied in the same way by the GPs in a real life situation. Pulse oximetry has so far not been frequently used in the assessment of exacerbations, although recommended in both the GOLD (Global Initiative for Chronic Obstructive Lung Disease) and GINA guidelines. In other settings, external factors such as short consultation times and the patient’s social circumstances influence prescribing behavior [33].

The inclusion of both asthma and COPD patients may be regarded as a weakness of the study. On the other hand, this mix of patients reflects real life in primary care, where it is often difficult to decide which of the two diseases the patient suffers from. FEV_1_/FVC ≥ 0.7 had been found in some patients in the COPD group at baseline, and FEV_1_/FVC < 0.7 in some patients in asthma group. Some symptoms typical for COPD were registered with almost similar frequency in both groups, such as increased phlegm and purulence. This indicates that the classification of patients into COPD and non-COPD groups is not crystal clear and that some patients with FEV_1_/FVC ≥ 0.7 suffered from an early COPD. In the COPD group more than half of the patients had also been given an asthma diagnosis the previous 5 years. COPD was most frequently the last diagnosis of the two [3], and there had been a trend of change in diagnosis from asthma to COPD since 1995 [34]. Asthma can develop into COPD [35], and a tendency to choose an asthma diagnosis when COPD could be questioned was strengthened by the reimbursement regulations for respiratory medication introduced in Norway in 2006. When the study was carried out, costs of inhaled corticosteroids combined with long-acting β_2_-agonists could be reimbursed, as a rule, only in patients with a diagnosis of asthma.

### Implications of the study

The GPs relied on the findings by physical examination, CRP and pulse oximetry more than on the patient’s respiratory symptoms when deciding type of treatment. While the usefulness of CRP is already strongly supported by recent evidence, the role of chest findings and oxygen saturation is not yet clear. Since GPs seems to heavily rely on chest findings in their treatment decisions, it will be interesting to evaluate their usefulness as clues for treatment for asthma and COPD in future studies. With more evidence available, it might be possible to decide whether or not the application of chest findings, CRP testing and pulse oximetry can lead to improved patient outcomes related to COPD and/or asthma exacerbations.

## Conclusion

In this study where CRP testing and pulse oximetry were carried out as routine, chest findings, together with the results of CRP and oxygen saturation were stronger predictors in choosing the type of treatment in patients suffering from exacerbation of asthma or COPD than were respiratory symptoms.

## Abbreviations

COPD: Chronic obstructive pulmonary disease
AECOPD: Acute exacerbation of chronic obstructive pulmonary disease
GP: General practitioner
CRP: C-reactive protein
FEV_1_: Forced expiratory volume in one second
FVC: Forces vital capacity
SpO2: Saturation of peripheral oxygen
